# Treatment with Ginger Ameliorates Fructose-Induced Fatty Liver and Hypertriglyceridemia in Rats: Modulation of the Hepatic Carbohydrate Response Element-Binding Protein-Mediated Pathway

**DOI:** 10.1155/2012/570948

**Published:** 2012-11-06

**Authors:** Huanqing Gao, Tao Guan, Chunli Li, Guowei Zuo, Johji Yamahara, Jianwei Wang, Yuhao Li

**Affiliations:** ^1^Faculty of Basic Medical Sciences, Chongqing Medical University, Chongqing 400016, China; ^2^Pharmafood Institute, Kyoto 602-8136, Japan; ^3^Endocrinology and Metabolism Group, Sydney Institute of Health Sciences, Sydney, NSW 2000, Australia

## Abstract

Ginger has been demonstrated to improve lipid derangements. However, its underlying triglyceride-lowering mechanisms remain unclear. Fructose overconsumption is associated with increase in hepatic de novo lipogenesis, thereby resulting in lipid derangements. Here we found that coadministration of the alcoholic extract of ginger (50 mg/kg/day, oral gavage, once daily) over 5 weeks reversed liquid fructose-induced increase in plasma triglyceride and glucose concentrations and hepatic triglyceride content in rats. Plasma nonesterified fatty acid concentration was also decreased. Attenuation of the increased vacuolization and Oil Red O staining area was evident on histological examination of liver in ginger-treated rats. However, ginger treatment did not affect chow intake and body weight. Further, ginger treatment suppressed fructose-stimulated overexpression of carbohydrate response element-binding protein (ChREBP) at the mRNA and protein levels in the liver. Consequently, hepatic expression of the ChREBP-targeted lipogenic genes responsible for fatty acid biosynthesis was also downregulated. In contrast, expression of neither peroxisome proliferator-activated receptor- (PPAR-) alpha and its downstream genes, nor PPAR-gamma and sterol regulatory element-binding protein 1c was altered. Thus the present findings suggest that in rats, amelioration of fructose-induced fatty liver and hypertriglyceridemia by ginger treatment involves modulation of the hepatic ChREBP-mediated pathway.

## 1. Introduction

Ginger (*Zingiber officinale* Roscoe, Zingiberacae), one of the most commonly used spices and medicinal plants around the world, has been found to have pleiotropic pharmacological activities, such as anti-inflammatory, antioxidant, and cardiovascular activities [1, 2]. It has been reported that ginger improves dietary (cholesterol, fructose, or high-fat diet) or streptozocin-induced lipid derangements in rodents [3–9]. It has been also demonstrated that modification of hepatic low density lipoprotein receptor and 3-hydroxy-3-methylglutaryl coenzyme A reductase expression is involved in improvement of cholesterol homeostasis [4, 8]. However, the underlying mechanisms of triglyceride-lowering effect of ginger remain unclear.

Strong evidence suggests that consumption of diets high in fructose results in fatty liver, dyslipidemia, insulin resistance, and obesity in animals and humans [10–12]. Fatty liver (excessive accumulation of triglyceride in hepatocytes) is the hallmark of nonalcoholic fatty liver disease, which has become an important public health problem due to its high prevalence, potential progression to severe liver disease, and association with cardiometabolic abnormalities [13–15]. Hypertriglyceridemia is a common dyslipidemia, that is, an independent risk of cardiovascular diseases [16]. 

In the present study, we tested the effects of ginger treatment on fructose-induced lipid derangements and investigated the underlying triglyceride-lowering mechanisms in rats.

## 2. Materials and Methods

### 2.1. Ginger Extract

Ginger rhizome was collected in the suburban area of Hanoi, Vietnam, and was identified botanically. The extract used in the present study was prepared using an ethanolic method. Briefly, 5 kg sliced ginger rhizomes including the skin were immersed in 5 L 95% ethanol with intermittent shaking for 24 h, then refluxed for 3 h by heating. The filtrate was evaporated under reduced pressure below 45°C. The residue (yield: 9.6%) was designated as an alcoholic extract. The extract was quantified by HPLC method described previously [17] to contain 2 representative components: 6-gingerol: 4.4% and 6-shogaol: 1.1%, respectively.

### 2.2. Animals, Diet and Experimental Protocol

 All animal procedures were in accordance with the “Principles of laboratory animal care” (http://grants1.nih.gov/grants/olaw/references/phspol.htm) and were approved by the Animal Ethics Committee, Chongqing Medical University, China.

Male Sprague-Dawley rats weighing 210–230 g and the standard chow (ingredients are shown in Table 1) were supplied by the laboratory animal center, Chongqing Medical University, China. Rats were housed in a temperature controlled facility (21 ± 1°C, 55 ± 5% relative humidity) with a 12 h light/dark cycle. Animals were allowed free access to water and the standard chow for at least 1 week prior to starting the experiments.

It is known that sugar-sweetened nonalcoholic beverages, such as soft drinks, appear as the major source of fructose for all classes of age considered, except for children younger than 6 year and adults older than 50 year [12]. Thus, fructose in drinking water was used in the present study as described previously [18–20]. In initial experiments, we found that compared to vehicle, ginger treatment significantly increased fructose intake when the rats had free access to 10% fructose in drinking water. In order to exclude the influence of the difference in intake of fructose, the sole pathogenic factor in the development of the adverse metabolic effects in this model, we adjusted the fructose consumption in ginger-treated rats to that of fructose controls. 24 rats were divided into 4 groups (*n* = 6 per group): (1) water control, free access to water; (2) fructose control, free access to 10% fructose solution (w/v, preparation every day); (3) fructose ginger 20 mg/kg; (4) fructose ginger 50 mg/kg, in which the fructose consumption was adjusted (by regulating the concentration of fructose solution) daily to that in the fructose-control group on the previous day. There was no difference in body weight between the groups before treatments were commenced. Animals in ginger-treated groups were administered ginger extract 20 and 50 mg/kg (suspended in 5% Gum Arabic solution, gavage once daily) for 5 weeks, respectively. The rats in the corresponding water- and fructose-control groups received vehicle (5% Gum Arabic) alone. All rats had free access to the standard chow. To avoid stress and more accurately monitor fructose intake, 2 rats were housed in a cage. The consumed chow and fructose solution were measured per 2 rats daily and the intake of fructose was calculated. On day 35, rats were deprived of chow, but still had free access to water (Group 1) or fructose solution (Group 2–4) for 14 h. Blood samples were collected by retroorbital venous puncture under either anesthesia at 9:00–12:00 am for determination of plasma concentrations of total cholesterol (kit from Kexin Institute of Biotechnology, Shanghai, China), triglyceride (Triglyceride-E kit, Wako, Osaka, Japan), non-esterified fatty acid (NEFA) (NEFA-C kit, Wako, Osaka, Japan), glucose (kit from Kexin Institute of Biotechnology, Shanghai, China), and insulin (kit from Morinaga Biochemical Industries, Tokyo, Japan). Immediately, animals were weighed and killed by prompt dislocation of the neck vertebra. Livers were collected and weighed. The ratio of liver weight to body weight was calculated. Segments of liver were snap frozen in liquid nitrogen and stored at −80°C for subsequent determination of gene/protein expression and triglyceride contents. 

### 2.3. Determination of Triglyceride Content in Liver

Triglyceride content in liver was determined as described previously [21]. Briefly, 100 mg of tissue was homogenized and extracted with 2 mL of isopropanol. After centrifugation (3000 rpm), the triglyceride content in supernatants was determined enzymatically (Wako, Osaka, Japan).

### 2.4. Histological Examination

A portion of liver was fixed with 10% formalin and embedded in paraffin. Four-micron sections were cut and stained with hematoxylin and eosin for examination of liver histology (BX-51, Olympus Corporation, Tokyo, Japan). To further confirm lipid droplet accumulation, six-micron frozen sections were stained with Oil Red O. Forty fields in three individual sections were randomly selected, and the Oil Red O-stained area and the total tissue area were measured using an ImageJ 1.43 analyzing system. The ratio of the Oil Red O-stained area to the total tissue area was calculated (%). 

### 2.5. Real-Time PCR

Total RNA was isolated from livers of individual rats using TRIzol (Takara, Dalian, China). cDNA was synthesized using M-MLV RTase cDNA Synthesis Kit (Takara, Dalian, China) according to the manufacturer's instructions. Real-time PCR was performed with the CFX 96 Real-Time PCR Detection System (Biorad Laboratories Inc., Hercules, CA, USA) using the SYBR Premix Ex Taq II (Takara, Dalian, China). The sequences of primers are shown in Table 2. The gene expression from each sample was analysed in duplicates and normalized against the internal control gene *β*-actin. Levels in water-control rats were arbitrarily assigned a value of 1.

### 2.6. Western Blot

Nuclear protein extracts were prepared from livers using the NE-PER nuclear and cytoplasmic extraction reagent kit (Pierce Biotechnology, Rockford, IL, USA), according to the manufacturer's instructions. Proteins from nuclear extracts (30 *μ*g) from livers were subjected to SDS-PAGE analysis on a 10% gel. Protein concentration was determined using the Bradford method (Bio Rad Laboratories, Hercules, CA, USA) using bovine serum albumin as a standard. Proteins were electrotransferred onto polyvinylidene fluoride membrane (Amersham, Buckinghamshire, UK). Carbohydrate response element-binding protein (ChREBP) was detected with a goat polyclonal antibody (dilution 1 : 200, Santa Cruz Biotechnology, Santa Cruz, CA, USA). Detection of signals was performed using the ECL western blot detection kit (Pierce Biotechnology, Rockford, IL, USA) with anti-goat horseradish peroxidase-conjugated IgG (dilution 1 : 5,000, Santa Cruz Biotechnology, Santa Cruz, CA, USA) as second antibody. Polyclonal rabbit Lamin A/C antibody (dilution 1 : 1000, Cell Signaling Technologies, Beverly, MA, USA) was used as loading control to normalize the signal obtained for nuclear ChREBP protein. The immunoreactive bands were visualized by autoradiography and the density was evaluated using ImageJ 1.43. Levels in water-control rats were arbitrarily assigned a value of 1.

### 2.7. Data Analysis

All results are expressed as means ± SEM. Data were analyzed by ANOVA using the StatView software, and followed by The Student Newman-Keuls test to locate the differences between groups. *P* < 0.05 was considered to be statistically significant.

## 3. Results

### 3.1. Fructose-Induced Adverse Effects in Rats

**Figure 1:**
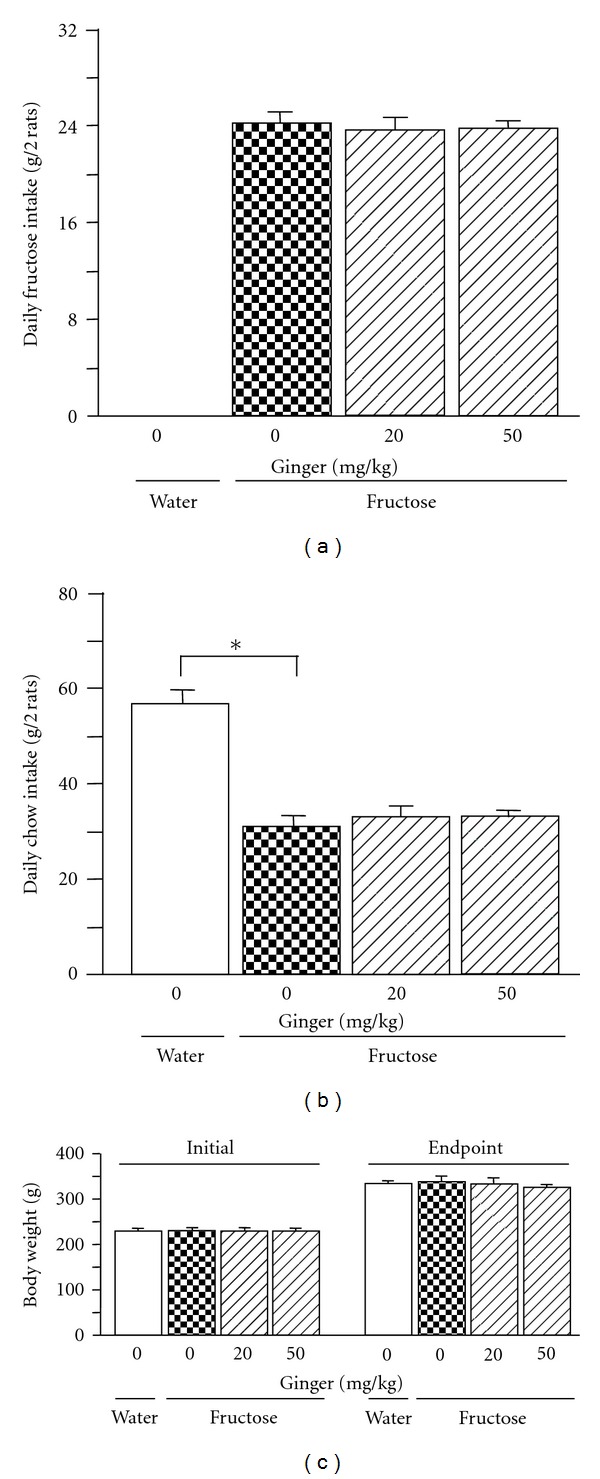
Intakes of fructose (a) and laboratory chow (b), and body weight (c) in water-control, 10% fructose solution-control, and fructose pair-fed ginger-treated rats. Animals were coadministered with ginger extract (20 or 50 mg/kg/day) or vehicle (ginger: 0 mg/kg, 5% Gum Arabic) by oral gavage daily for 5 weeks. Data are means ± SEM (*n* = 6 each group). *P < 0.05.

Compared to water drinking, intake of 10% fructose solution decreased intake of chow (Figure 1(b)), but did not affect body weight (Figure 1(c)). 

**Figure 2:**
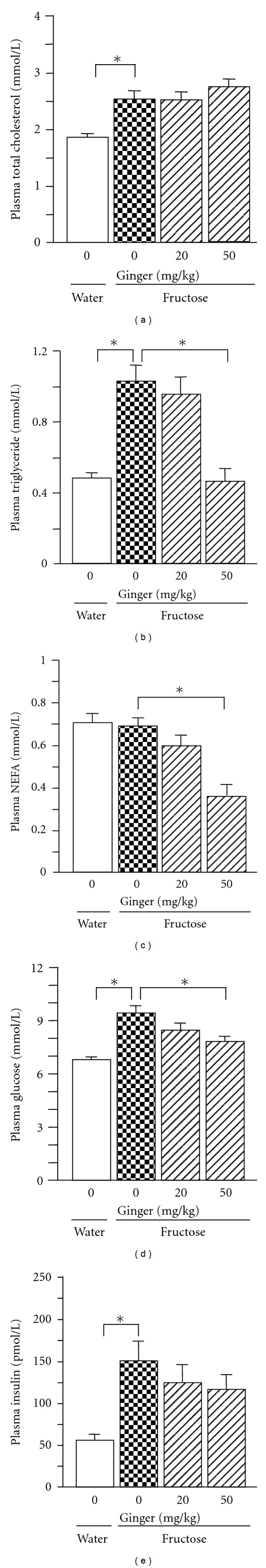
Plasma total cholesterol (a), triglyceride (b), NEFA (c), glucose (d), and insulin (e) concentrations in water-control, 10% fructose solution-control, and fructose pair-fed ginger-treated rats at week 5. Animals were coadministered with ginger extract (20 or 50 mg/kg/day) or vehicle (ginger: 0 mg/kg, 5% Gum Arabic) by oral gavage daily for 5 weeks. Data are means ± SEM (*n* = 6 each group). *P < 0.05.

Under the status of feeding fructose solution, plasma concentrations of total cholesterol (Figure 2(a)), triglyceride (Figure 2(b)), glucose (Figure 2(d)), and insulin (Figure 2(e)) were elevated, whereas plasma NEFA concentration (Figure 2(c)) was unchanged.

**Figure 3:**
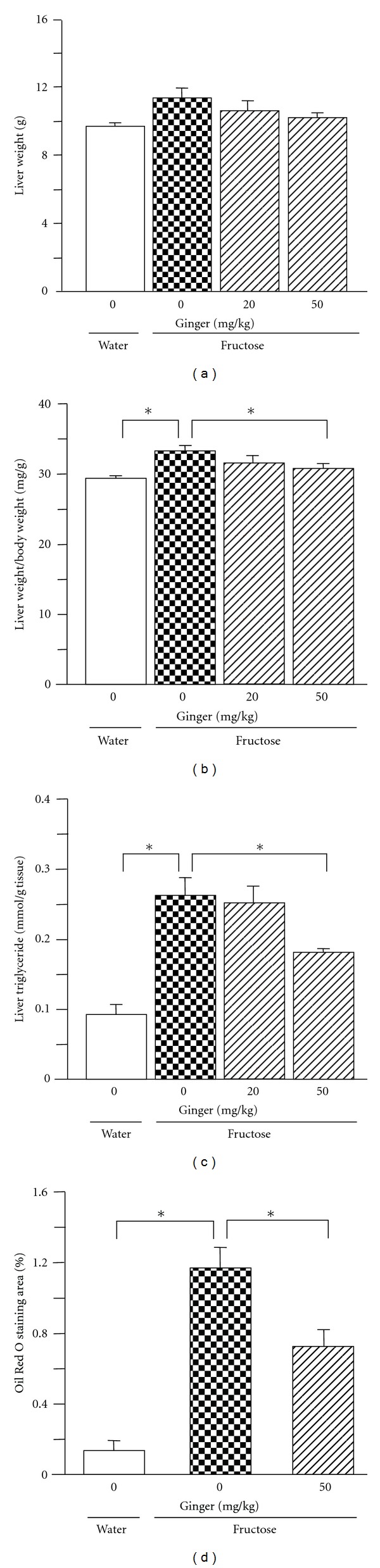
Liver weight (a), the ratio of liver weight to body weight (b), hepatic triglyceride content (c), and Oil Red O staining area (d) in water-control, 10% fructose solution-control, and fructose pair-fed ginger-treated rats at week 5. Animals were coadministered with ginger extract (20 or 50 mg/kg/day) or vehicle (ginger: 0 mg/kg, 5% Gum Arabic) by oral gavage daily for 5 weeks. Data are means ± SEM (*n* = 6 each group). *P < 0.05.

**Figure 4:**
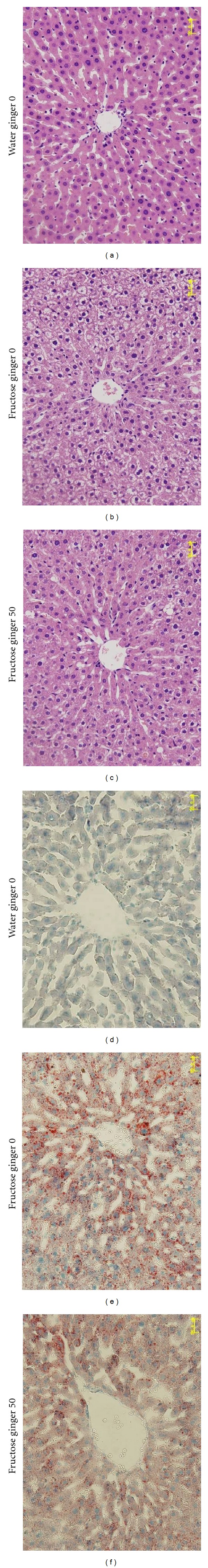
Representative images showing histology of liver (hematoxylin and eosin-staining, (a)–(c); Oil Red O staining, (d)–(f). X200) in water-control, 10% fructose solution-control, and fructose pair-fed ginger-treated rats at week 5. Animals were coadministered with ginger extract (50 mg/kg/day) or vehicle (ginger: 0 mg/kg, 5% Gum Arabic) by oral gavage daily for 5 weeks. Data are means ± SEM (*n* = 6 each group). *P < 0.05.

Although fructose feeding did not significantly affect liver weight (Figure 3(a)), the ratio of liver weight to body weight (Figure 3(b)) and hepatic triglyceride content were increased (Figure 3(c)). In accord with this finding, increased vacuolization (Figure 4(b)) and Oil Red O staining area (Figures 3(d) and 4(e)) were evident on histological examination of liver sections from fructose-fed rats compared with water-control rats (Figures 3(d), 4(a) and 4(d)), indicative of excess lipid droplet accumulation. 

### 3.2. Effects of Ginger Treatment in Fructose-Fed Rats

Ginger treatments did not affect intake of chow (Figure 1(b)), body weight (Figure 1(c)), plasma total cholesterol (Figure 2(a)), and liver weight (Figure 3(a)). However, plasma triglyceride (Figure 2(b)), NEFA (Figure 2(c)) and glucose (Figure 2(d)) concentrations, and the ratio of liver weight to body weight (Figure 3(b)) and hepatic triglyceride content (Figure 3(c)) were decreased after treatment with ginger at 50 mg/kg. Plasma insulin concentration (Figure 2(e)) also had a trend to decrease. Vacuolization (Figure 4(c)) and Oil Red O staining area (Figures 3(d) and 4(f)) in liver were also reduced. Low dosage of ginger extract showed less effect.

### 3.3. Hepatic Gene/Protein Expression Profiles in Fructose-Fed Rats

**Figure 5:**
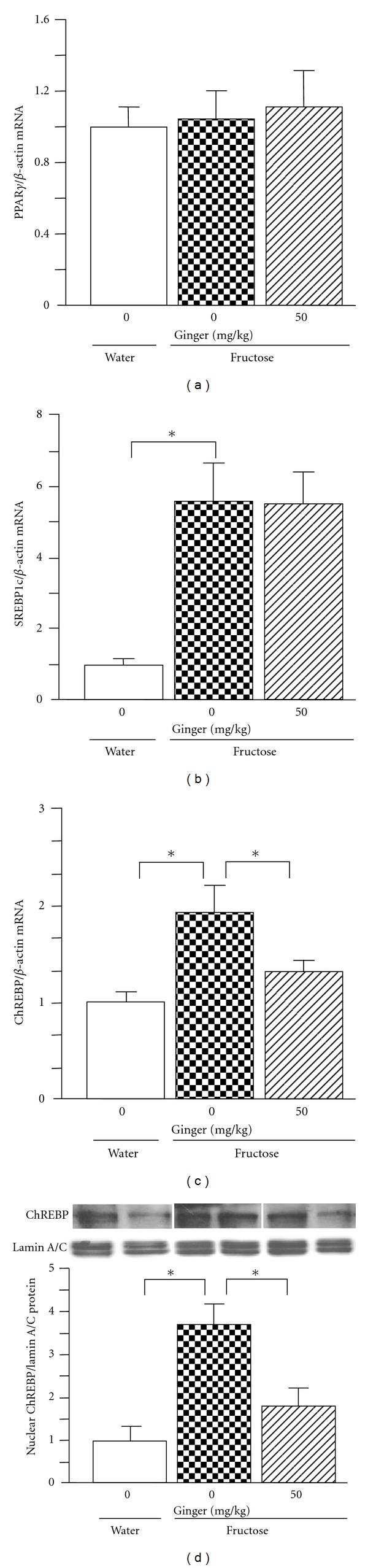
Hepatic mRNA expression of PPAR-*γ* (a), sterol regulatory element-binding protein (SREBP)1c (b), carbohydrate response element-binding protein (ChREBP) (c), and nuclear ChREBP protein expression (d) in water-control, 10% fructose solution-control, and fructose pair-fed ginger-treated rats at week 5. Animals were coadministered with ginger extract (50 mg/kg/day) or vehicle (ginger: 0 mg/kg, 5% Gum Arabic) by oral gavage daily for 5 weeks. mRNA was determined by real-time PCR. Protein expression was determined by western blot. Data are means ± SEM (*n* = 6 each group). *P < 0.05.

**Figure 6:**
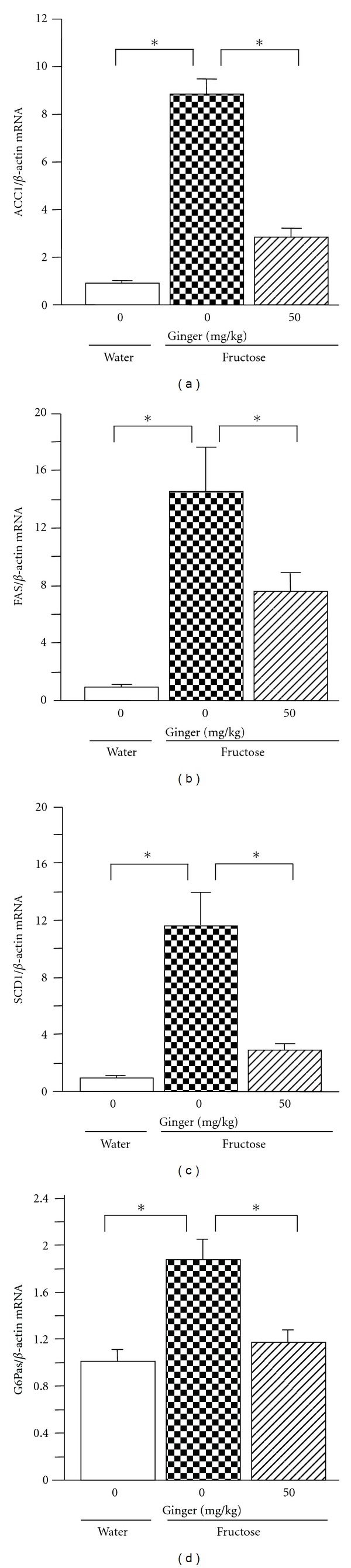
Hepatic mRNA expression of acetyl-CoA carboxylase (ACC)1 (a), fatty acid synthase (FAS) (b), stearoyl-CoA desaturase (SCD)1 (c), and glucose-6-phosphatase (G6Pase) (d) in water-control, 10% fructose solution-control, and fructose pair-fed ginger-treated rats at week 5. Animals were coadministered with ginger extract (50 mg/kg/day) or vehicle (ginger: 0 mg/kg, 5% Gum Arabic) by oral gavage daily for 5 weeks. mRNA was determined by real-time PCR. Data are means ± SEM (*n* = 6 each group). *P < 0.05.

By real-time PCR, fructose feeding did not alter hepatic expression of peroxisome proliferator-activated receptor-(PPAR-) *γ* (Figure 5(a)). However, mRNA levels of sterol regulatory element-binding protein (SREBP)1c (Figure 5(b)), ChREBP (Figure 5(c)), acetyl-CoA carboxylase (ACC)1 (Figure 6(a)), fatty acid synthase (FAS) (Figure 6(b)), stearoyl-CoA desaturase (SCD)1 (Figure 6(c)), and glucose-6-phosphatase (G6Pase) (Figure 6(d)) were increased substantially. The increase in nuclear ChREBP protein content was demonstrated by western blot analysis (Figure 5(d)). After ginger treatment (50 mg/kg), pronounced suppression of mRNAs encoding ChREBP, ACC1, FAS, SCD1, and G6Pase was noted. The increase in nuclear ChREBP protein expression was also downregulated. However, ginger treatment altered neither PPAR-*γ* nor SREBP1c mRNA expression.

**Figure 7:**
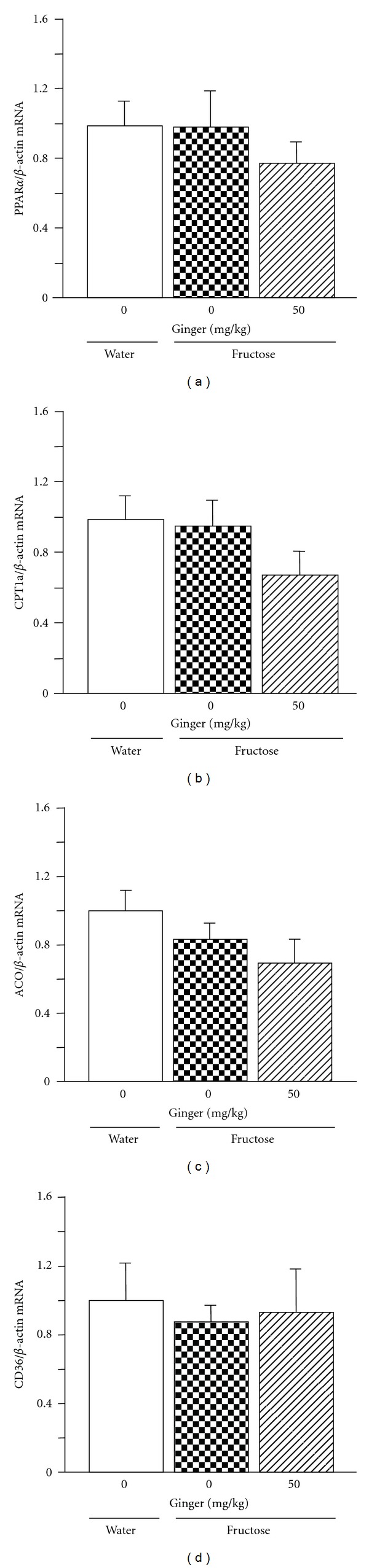
Hepatic mRNA expression of peroxisome proliferator-activated receptor-(PPAR-) *α* (a), carnitine palmitoyltransferase (CPT)1a (b), acyl-CoA oxidase (ACO) (c), and CD36 (d) in water-control, 10% fructose solution-control, and fructose pair-fed ginger-treated rats at week 5. Animals were coadministered with ginger extract (50 mg/kg/day) or vehicle (ginger: 0 mg/kg, 5% Gum Arabic) by oral gavage daily for 5 weeks. mRNA was determined by real-time PCR. Data are means ± SEM (*n* = 6 each group). *P < 0.05.

Also in liver, fructose feeding did not affect PPAR-*α* (Figure 7(a)), carnitine palmitoyltransferase (CPT)1a (Figure 7(b)), acyl-CoA oxidase (ACO) (Figure 7(c)), and CD36 (Figure 7(d)) gene expression. Ginger treatment was also without effect on mRNA levels of these genes in fructose-fed rats.

## 4. Discussion

The present findings demonstrate that treatment of rats with ginger extract ameliorates long-term fructose consumption-induced fatty liver and hypertriglyceridemia, accompanied by a decrease in plasma glucose concentration.

Studies in humans and in rodents have demonstrated that an increase in hepatic de novo lipid synthesis plays a pivotal role in excessive fat accumulation in liver [14]. Fructose, by providing large amounts of hepatic triose-phosphate as precursors for fatty acid synthesis, is highly lipogenic [12]. Recent findings suggest that increase in hepatic de novo lipogenesis plays an important role in fructose feeding-induced fatty liver and hypertriglyceridemia [11, 12]. In the present study, treatment with ginger extract did not affect intake of chow, but reversed the upregulated hepatic mRNA levels of ACC1, FAS, and SCD1, the genes responsible for de novo fatty acid synthesis. Thus, these results suggest that ginger treatment suppresses the increased hepatic de novo lipogenesis.

De novo hepatic lipogenesis is mediated by two important proteins, ChREBP and SREBP1c [12, 14, 22]. ChREBP, a transcriptional regulator of glucose and lipid metabolism, is an attractive target for lipid-lowering therapies in obesity and diabetes [23]. ChREBP plays a critical role in converting excess carbohydrates into triglycerides. Liver-specific inhibition of ChREBP improves hepatic steatosis, hypertriglyceridemia, and insulin resistance, accompanied by downregulation of hepatic expression of lipogenic and gluconeogenetic genes, including those encoding ACC, FAS SCD1, and G6Pase in ob/ob mice [24, 25]. Fructose administration activates ChREBP which acts in synergy with SREBP to increase the expression of lipogenic genes [12, 26–28]. Incubation of HepG2 cells with fructose also induces upregulation of nuclear ChREBP and SREBP1c protein expression [29]. In contrast, SREBP1c is stimulated by insulin [14]. Although SREBP1c plays a major role in the long-term control of glucose and lipid homeostasis by insulin, SREBP1c activity alone does not appear to fully account for the stimulation of glycolytic and lipogenic gene expression in response to carbohydrate diet [23]. In the present study, hepatic mRNA expression of both ChREBP and SREBP1c in fructose-fed rats was upregulated. The increase in nuclear ChREBP was confirmed at the protein level. Treatment with ginger extract abolished the ChREBP overexpression, accompanied by downregulation of expression of its targeted lipogenic and gluconeogenetic genes ACC1, FAS, SCD1, and G6Pas. However, the increased SREBP1c expression was unchanged. Taken together, these findings suggest that modulation of the ChREBP-mediated pathway is responsible for ginger treatment-elicited improvement of fatty liver and hypertriglyceridemia. It needs to further investigate why and how ginger selectively works on ChREBP, but not SREBP1c.

PPAR-*γ* is a member of the ligand-activated nuclear receptor superfamily and predominantly expressed in adipose tissue and normally at low level in liver [30]. Pharmacologic activation of PPAR-*γ* upregulates the genes encoding molecules that promote a combination of lipid storage and lipogenesis, such as CD36, SREBP1, and SCD1 [30]. Activation of this metabolic pathway causes body-wide lipid repartitioning by increasing the triglyceride content of adipose tissue and lowering free fatty acids and triglycerides in the circulation, liver, and muscle, thereby improving insulin sensitivity [30]. In mice, activation of PPAR-*γ* in liver appears to contribute to the development of hepatic steatosis [31, 32]. In rats, however, fructose feeding [20], high-fat diet feeding [33], combination of high-fat diet feeding and streptozotocin [34], or leptin receptor mutation [35] cause excess lipid accumulation in liver, but does not increase hepatic PPAR-*γ* expression. These findings may suggest the difference in hepatic PPAR-*γ* expression between animal species. It has been demonstrated that 6-shogaol acts as a PPAR-*γ* agonist in 3T3-L1 adipocytes derived from mice [36]. In the present study, fructose feeding did not change hepatic expression of PPAR-*γ* and its responsive gene CD36 in rats. Furthermore, hepatic SREBP1c and SCD1 mRNAs were substantially upregulated after fructose consumption. Treatment of ginger extract did not alter hepatic expression of PPAR-*γ*, SREBP1c, and CD36, but markedly suppressed the SCD1 overexpression in fructose-fed rats. Thus, our findings in gene expression do not support the involvement of the hepatic PPAR-*γ* pathway in the triglyceride-lowering effect of ginger treatment. It still needs to further investigate whether ginger treatment modulates adipose PPAR-*γ*-mediated gene expression and activities in fructose-fed rats.

In contrast to PPAR-*γ*, PPAR-*α*, predominantly expressed in liver and to a lesser extent in heart and muscle, controls lipid metabolism and glucose homeostasis in liver and skeletal muscle [30, 37]. PPAR-*α* influences intracellular lipid and carbohydrate metabolism through direct transcriptional control of the genes involved in peroxisomal and mitochondrial *β*-oxidation pathways, fatty acid uptake, and triglyceride catabolism, such as CPT1a, ACO, and CD36 [30, 37]. However, lipid disposal via fatty acid *β*-oxidation only slightly affects hepatic triglyceride accumulation [14]. The relationship between fructose feeding and hepatic expression of PPAR-*α* and its responsive genes is still controversial. Some studies have reported that fructose feeding downregulated hepatic expression of PPAR-*α*, CPT1a, and/or ACO genes [18–20, 38], whereas others found no change in the expression of these genes [28, 29, 39–41]. It has been demonstrated that activation of PPAR-*α* by its agonist fenofibrate strongly induced the expression of hepatic lipogenic genes FAS, ACC1, and SCD1, accompanied by induction of hepatic CPT1a, ACO, and CD36 in mice [42, 43]. SCD1 is a direct target for PPAR-*α* and is activated by the PPAR-*α* agonists clofibrate and gemfibrozil [44] and fenofibrate [43]. In addition, hepatomegaly (an increase in liver weight) is a well-known important marker of activation of PPAR-*α* in rodents [30]. In the present study, 5-week fructose feeding did not alter hepatic PPAR-*α*-responsive gene expression. There was also no significant difference in the expression of these genes between fructose vehicle and fructose ginger-treated groups. Furthermore, ginger treatment decreased liver weight/the ratio of liver weight to body weight. Thus, our findings do not support the involvement of the hepatic PPAR-*α* pathway in the effects of ginger treatment.

Increased fat delivery from peripheral fats stored in white adipose tissue is also largely associated with excessive fat accumulation in liver [14]. Recent evidence suggests that adipose tissue insulin resistance is closely correlated with metabolic parameters and hepatic histological damage in patients with nonalcoholic steatohepatitis; amelioration of adipose tissue insulin resistance may contribute to the improvement of metabolic derangements and hepatic injuries [45–47]. In the setting of insulin resistance, insulin is unable to properly suppress lipolysis in adipose tissue, resulting in a relative increase in free fatty acid release into the plasma [48]. It has been demonstrated that 10% fructose in drinking water for 2 weeks did not increase plasma NEFA concentration in rats [18]. In contrast, a diet containing 66.8% [49] or 50% [28] fructose for 4 weeks significantly increased plasma NEFA concentration in rats. These findings suggest that fructose overconsumption may induce adipose tissue insulin resistance, thereby increasing release of fatty acids to circulation, then to liver. In the present study, plasma NEFA concentration was not altered in the rats that were deprived of chow, but still had free access to 10% fructose in drinking water, compared to the rats that had free access to water. Ginger treatment significantly decreased plasma NEFA concentration. These results suggest the possibility that ginger treatment may modulate lipolysis in adipose tissue. However, fructose intake in the present study might interfere with fatty acid release from adipose tissue. It has been suggested that analysis of both plasma fatty acid changes during the oral glucose tolerance test assessment and the adipose tissue insulin resistance index is suitable for evaluation of insulin action in adipose tissues [45–47]. It will be very interesting to further investigate whether the adipose pathway also contributes to fructose consumption-induced fatty liver and the hepatoprotective effect of ginger treatment.

The constituents of ginger are numerous. Although ginger has been utilized in many studies in both humans and animals, there is a relative dearth of information on its bioavailability. [6]-gingerol and [6]-shogaol (the latter is a dehydrated form of gingerols), two of the major components contained in the crude materials, have been implicated in most of the pharmacological activities of ginger [2]. The findings in rats suggest that [6]-gingerol is metabolized partly in the liver, and to a much lesser extent, in the kidneys [2]. In the present study, ginger treatment ameliorated fructose-induced fatty liver and hypertriglyceridemia and suppressed fructose-stimulated hepatic overexpression of ChREBP-targeted genes in rats. It needs to further investigate how ginger extract modifies hepatic genes and whether [6]-gingerol is responsible for the metabolic effects of ginger observed in the present study.

Taken together, our present findings demonstrate that treatment with the ethanolic extract of ginger ameliorates fructose-induced fatty liver and hypertriglyceridemia in rats, which involves modulation of the hepatic ChREBP-mediated pathway.

## Figures and Tables

**Table 1:** Composition of the laboratory chow.

Ingredient	Unit	Content
Crude protein	%	18.50
Crude oil	%	4.00
Crude fibre	%	5.00
Crude ash	%	7.50
Moisture	%	10.00
Calcium	%	1.20
Phosphorous	%	0.60
Salt	%	0.85
Magnesium	%	0.20
Copper	mg/kg	10.00
Iron	mg/kg	100.00
Zinc	mg/kg	40.00
Vitamin A	(iu/Kg)	8000.00
Vitamin K	mg/kg	5.00
Nicotinic acid	mg/kg	45.00
Pantothenic acid	mg/kg	20.00
Vitamin D	(iu/Kg)	1000.00
Vitamin E	(iu/Kg)	60.00
Riboflavin	mg/kg	10.00
Iodine	mg/kg	1.00
Methionine + cystine	%	0.54
Threonine	%	0.65
Vitamin B12	mg/kg	0.02
Thiamin	mg/kg	8.00
Selenium	mg/kg	0.10

**Table 2:** Primer sequences for real-time PCR assays.

Gene	Forward primers	Reverse primers
*β*-actin	ACGGTCAGGTCATCACTATCG	GGCATAGAGGTCTTTACGGATG
PPAR*γ*	GCCCTTTGGTGACTTTATGGAG	GCAGCAGGTTGTCTTGGATGT
SREBP1c	CTGTCGTCTACCATAAGCTGCAC	ATAGCATCTCCTGCACACTCAGC
ChREBP	GAAGACCCAAAGACCAAGATGC	TCTGACAACAAAGCAGGAGGTG
ACC1	AACATCCCGCACCTTCTTCTAC	CTTCCACAAACCAGCGTCTC
FAS	ACCTCATCACTAGAAGCCACCAG	GTGGTACTTGGCCTTGGGTTTA
SCD1	CAGTTCCTACACGACCACCACTA	GGACGGATGTCTTCTTCCAGAT
G6Pase	GAGTGGCTCAACCTCGTCTTC	AAGGGAACTGGTGAATCTGGAC
PPAR*α*	GTCATCACAGACACCCTCTCTCC	TGTCCCCACATATTCGACACTC
CPT1a	CTGCTGTATCGTCGCACATTAG	GTTGGATGGTGTCTGTCTCTTCC
ACO	CCCAAGACCCAAGAGTTCATTC	TCACGGATAGGGACAACAAAGG
CD36	AACCCAGAGGAAGTGGCAAAG	GACAGTGAAGGCTCAAAGATGG

Sequences: 5′ to 3′.

## References

[1] Nicoll R, Henein MY (2009). Ginger (*Zingiber officinale* Roscoe): a hot remedy for cardiovascular disease?. *International Journal of Cardiology*.

[2] Ali BH, Blunden G, Tanira MO, Nemmar A (2008). Some phytochemical, pharmacological and toxicological properties of ginger (*Zingiber officinale* Roscoe): a review of recent research. *Food and Chemical Toxicology*.

[3] Bhandari U, Sharma JN, Zafar R (1998). The protective action of ethanolic ginger (*Zingiber officinale*) extract in cholesterol fed rabbits. *Journal of Ethnopharmacology*.

[4] Fuhrman B, Rosenblat M, Hayek T, Coleman R, Aviram M (2000). Ginger extract consumption reduces plasma cholesterol, inhibits LDL oxidation and attenuates development of atherosclerosis in atherosclerotic, apolipoprotein E-deficient mice. *Journal of Nutrition*.

[5] Kadnur SV, Goyal RK (2005). Beneficial effects of *Zingiber officinale* Roscoe on fructose induced hyperlipidemia and hyperinsulinemia in rats. *Indian Journal of Experimental Biology*.

[6] Al-Amin ZM, Thomson M, Al-Qattan KK, Ali M (2006). Anti-diabetic and hypolipidaemic properties of ginger (*Zingiber officinale*) in streptozotocin-induced diabetic rats. *British Journal of Nutrition*.

[7] Nammi S, Sreemantula S, Roufogalis BD (2009). Protective effects of ethanolic extract of *Zingiber officinale* rhizome on the development of metabolic syndrome in high-fat diet-fed rats. *Basic and Clinical Pharmacology and Toxicology*.

[8] Nammi S, Kim MS, Gavande NS, Li GQ, Roufogalis BD (2010). Regulation of low-density lipoprotein receptor and 3-hydroxy-3-methylglutaryl coenzyme A reductase expression by *Zingiber officinale* in the liver of high-fat diet-fed rats. *Basic and Clinical Pharmacology and Toxicology*.

[9] Beattie JH, Nicol F, Gordon M-J (2011). Ginger phytochemicals mitigate the obesogenic effects of a high-fat diet in mice: a proteomic and biomarker network analysis. *Molecular Nutrition and Food Research*.

[10] Johnson RJ, Perez-Pozo SE, Sautin YY (2009). Hypothesis: Could excessive fructose intake and uric acid cause type 2 diabetes?. *Endocrine Reviews*.

[11] Stanhope KL, Schwarz JM, Keim NL (2009). Consuming fructose-sweetened, not glucose-sweetened, beverages increases visceral adiposity and lipids and decreases insulin sensitivity in overweight/obese humans. *Journal of Clinical Investigation*.

[12] Tappy L, Lê KA (2010). Metabolic effects of fructose and the worldwide increase in obesity. *Physiological Reviews*.

[13] Marchesini G, Bugianesi E, Forlani G (2003). Nonalcoholic fatty liver, steatohepatitis, and the metabolic syndrome. *Hepatology*.

[14] Postic C, Girard J (2008). Contribution of de novo fatty acid synthesis to hepatic steatosis and insulin resistance: lessons from genetically engineered mice. *Journal of Clinical Investigation*.

[15] Abdelmalek MF, Suzuki A, Guy C (2010). Increased fructose consumption is associated with fibrosis severity in patients with nonalcoholic fatty liver disease. *Hepatology*.

[16] Brunzell JD (2007). Hypertriglyceridemia. *New England Journal of Medicine*.

[17] Rong X, Peng G, Suzuki T, Yang Q, Yamahara J, Li Y (2009). A 35-day gavage safety assessment of ginger in rats. *Regulatory Toxicology and Pharmacology*.

[18] Roglans N, Vilà L, Farré M (2007). Impairment of hepatic STAT-3 activation and reduction of PPAR*α* activity in fructose-fed rats. *Hepatology*.

[19] Huang XS, Zhao SP, Bai L, Hu M, Zhao W, Zhang Q (2009). Atorvastatin and fenofibrate increase apolipoprotein AV and decrease triglycerides by up-regulating peroxisome proliferator-activated receptor-*α*. *British Journal of Pharmacology*.

[20] Roglans N, Sanguino E, Peris C (2002). Atorvastatin treatment induced peroxisome proliferator-activated receptor *α* expression and decreased plasma nonesterified fatty acids and liver triglyceride in fructose-fed rats. *Journal of Pharmacology and Experimental Therapeutics*.

[21] Rong X, Li Y, Ebihara K (2010). Angiotensin II type 1 receptor-independent beneficial effects of telmisartan on dietary-induced obesity, insulin resistance and fatty liver in mice. *Diabetologia*.

[22] Denechaud PD, Dentin R, Girard J, Postic C (2008). Role of ChREBP in hepatic steatosis and insulin resistance. *FEBS Letters*.

[23] Postic C, Dentin R, Denechaud PD, Girard J (2007). ChREBP, a transcriptional regulator of glucose and lipid metabolism. *Annual Review of Nutrition*.

[24] Dentin R, Benhamed F, Hainault I (2006). Liver-specific inhibition of ChREBP improves hepatic steatosis and insulin resistance in ob/ob mice. *Diabetes*.

[25] Iizuka K, Miller B, Uyeda K (2006). Deficiency of carbohydrate-activated transcription factor ChREBP prevents obesity and improves plasma glucose control in leptin-deficient (ob/ob) mice. *American Journal of Physiology*.

[26] Uyeda K, Repa JJ (2006). Carbohydrate response element binding protein, ChREBP, a transcription factor coupling hepatic glucose utilization and lipid synthesis. *Cell Metabolism*.

[27] Koo HY, Miyashita M, Simon Cho BH, Nakamura MT (2009). Replacing dietary glucose with fructose increases ChREBP activity and SREBP-1 protein in rat liver nucleus. *Biochemical and Biophysical Research Communications*.

[28] Rodríguez-Calvo R, Barroso E, Serrano L (2009). Atorvastatin prevents carbohydrate response element binding protein activation in the fructose-fed rat by activating protein kinase A. *Hepatology*.

[29] Mori T, Kondo H, Hase T, Murase T (2011). Dietary phospholipids ameliorate fructose-induced hepatic lipid and metabolic abnormalities in rats. *Journal of Nutrition*.

[30] Evans RM, Barish GD, Wang YX (2004). PPARs and the complex journey to obesity. *Nature Medicine*.

[31] Chao L, Marcus-Samuels B, Mason MM (2000). Adipose tissue is required for the antidiabetic, but not for the hypolipidemic, effect of thiazolidinediones. *Journal of Clinical Investigation*.

[32] Gavrilova O, Haluzik M, Matsusue K (2003). Liver peroxisome proliferator-activated receptor *γ* contributes to hepatic steatosis, triglyceride clearance, and regulation of body fat mass. *Journal of Biological Chemistry*.

[33] Kang SA, Hong K, Jang KH, Kim YY, Choue R, Lim Y (2006). Altered mRNA expression of hepatic lipogenic enzyme and PPAR*α* in rats fed dietary levan from Zymomonas mobilis. *Journal of Nutritional Biochemistry*.

[34] Sharma AK, Bharti S, Ojha S (2011). Up-regulation of PPAR*γ*, heat shock protein-27 and -72 by naringin attenuates insulin resistance, *β*-cell dysfunction, hepatic steatosis and kidney damage in a rat model of type 2 diabetes. *British Journal of Nutrition*.

[35] Xu KZY, Zhu C, Kim MS, Yamahara J, Li Y (2009). Pomegranate flower ameliorates fatty liver in an animal model of type 2 diabetes and obesity. *Journal of Ethnopharmacology*.

[36] Isa Y, Miyakawa Y, Yanagisawa M (2008). 6-Shogaol and 6-gingerol, the pungent of ginger, inhibit TNF-*α* mediated downregulation of adiponectin expression via different mechanisms in 3T3-L1 adipocytes. *Biochemical and Biophysical Research Communications*.

[37] Lefebvre P, Chinetti G, Fruchart JC, Staels B (2006). Sorting out the roles of PPAR*α* in energy metabolism and vascular homeostasis. *Journal of Clinical Investigation*.

[38] Nagai Y, Nishio Y, Nakamura T, Maegawa H, Kikkawa R, Kashiwagi A (2002). Amelioration of high fructose-induced metabolic derangements by activation of PPAR*α*. *American Journal of Physiology*.

[39] Qu S, Su D, Altomonte J (2007). PPAR*α* mediates the hypolipidemic action of fibrates by antagonizing FoxO1. *American Journal of Physiology*.

[40] Yokozawa T, Hyun JK, Eun JC (2008). Gravinol ameliorates high-fructose-induced metabolic syndrome through regulation of lipid metabolism and proinflammatory state in rats. *Journal of Agricultural and Food Chemistry*.

[41] Kim HY, Okubo T, Juneja LR, Yokozawa T (2010). The protective role of amla (*Emblica officinalis* Gaertn.) against fructose-induced metabolic syndrome in a rat model. *British Journal of Nutrition*.

[42] Oosterveer MH, Grefhorst A, van Dijk TH (2009). Fenofibrate simultaneously induces hepatic fatty acid oxidation, synthesis, and elongation in mice. *Journal of Biological Chemistry*.

[43] Bijland S, Pieterman EJ, Maas ACE (2010). Fenofibrate increases very low density lipoprotein triglyceride production despite reducing plasma triglyceride levels in APOE∗3-Leiden.CETP mice. *Journal of Biological Chemistry*.

[44] Miller CW, Ntambi JM (1996). Peroxisome proliferators induce mouse liver stearoyl-CoA desaturase 1 gene expression. *Proceedings of the National Academy of Sciences of the United States of America*.

[45] Gastaldelli A, Harrison SA, Belfort-Aguilar R (2009). Importance of changes in adipose tissue insulin resistance to histological response during thiazolidinedione treatment of patients with nonalcoholic steatohepatitis. *Hepatology*.

[46] Neuschwander-Tetri BA (2010). Hepatic lipotoxicity and the pathogenesis of nonalcoholic steatohepatitis: the central role of nontriglyceride fatty acid metabolites. *Hepatology*.

[47] Lomonaco R, Ortiz-Lopez C, Orsak B (2012). Effect of adipose tissue insulin resistance on metabolic parameters and liver histology in obese patients with nonalcoholic fatty liver disease. *Hepatology*.

[48] Eckel RH, Grundy SM, Zimmet PZ (2005). The metabolic syndrome. *Lancet*.

[49] Nagai Y, Yonemitsu S, Erion DM (2009). The role of peroxisome proliferator-activated receptor *γ*
coactivator-1 *β* in the pathogenesis of fructose-induced insulin resistance. *Cell Metabolism*.

